# Metformin alleviates neurocognitive impairment in aging via activation of AMPK/BDNF/PI3K pathway

**DOI:** 10.1038/s41598-022-20945-7

**Published:** 2022-10-12

**Authors:** Omnia Ameen, Rehab M. Samaka, Reda A. A. Abo-Elsoud

**Affiliations:** 1 Clinical Physiology Department, Faculty of Medicine, Menoufia University, Menoufia, Egypt; 2 Pathology Department, Faculty of Medicine, Menoufia University, Menoufia, Egypt

**Keywords:** Physiology, Neurology

## Abstract

Slowing down age-related neurocognitive impairment has been a challenge. We evaluated the therapeutic effects of metformin in d-galactose-induced aging. Additionally, we studied the potential molecular mechanisms that could be responsible for metformin's anti-aging effects. Thirty male rats were equally divided into: 1—control group, which received saline solution, 2—d-galactose (D-gal) group, which received d-galactose (100 mg/kg/day) by gastric lavage for eight weeks, and 3—d-galactose + Metformin (D-gal + Met) treated group, which received d-galactose + metformin (200 mg/kg/day) by gastric lavage for eight weeks. Neurocognitive assessment was done. Measurement of inflammatory, oxidative stress, and BDNF biomarkers was performed. AMPK and PI3K genes expression were assessed. Hippocampal tissues were dissected for histopathological and immunohistochemical studies. D-gal resulted in neurocognitive impairments, elevation of inflammatory biomarkers, altered oxidative stress markers, decreased BDNF, decreased expression of synaptophysin and Bcl2 with increased expression of Caspase-3, and down-regulation of AMPK and PI3K genes. Neurodegenerative changes were present in the hippocampus. Metformin restored significantly D-gal induced neurodegenerative changes. We concluded that metformin could alleviate age-induced neurocognitive deficit via amelioration of neuroinflammation, attenuation of oxidative stress, reduction of apoptosis, as well as promotion of synaptic plasticity. These mechanisms could be mediated via the activation of the AMPK/BDNF/PI3K pathway.

## Introduction

The steady loss of physiological function, mental agility, and memory that occurs with aging is a multifaceted process that may be triggered by neuronal cell death^1^. The life expectancy in many countries is projected to exceed 85 years by 2030^2^. Because of the decreased quality of life for the elderly and the associated economical and personal expenditures, aging-related cognitive impairments have emerged as a significant social issue. In addition, brain aging is the primary cause of the development of neurodegenerative diseases which frequently progress irreversibly^3^. There are few or no effective treatments currently available for aging-related neurocogitive impairment^4^.

d-galactose (D-gal), a monosaccharide abundant in milk products, is normally converted to glucose by enzymes. However, long-term systemic D-gal administration can pose substantial health hazards^5^. Previous research suggested that persistent systemic D-gal delivery to rats could transform them into models of aging and age-related neurodegenerative disorders^6^. The D-gal aging model has several advantages over the natural aging model, including the ability to concentrate solely on the aging process. In the natural aging model, comorbidities including diabetes and hypertension are complicating variables^7^.

D-gal is thought to generate biochemical anomalies similar to those seen in the aging human brain, such as reduced the activity of antioxidant enzymes and attenuation of the cholinergic neurons. Advanced glycation end products, which are linked to both normal aging and the pathophysiology of many diseases, are produced by interaction of D-gal with protein amino groups. Animals provided D-gal caused neuroinflammation, mitochondrial damage, and oxidative damage from the development of Reactive Oxygen Species (ROS) along with problems in novelty habituation, deficiencies in spatial learning, and cognitive functions^8^.

Inflammation could be a key factor in cognitive deterioration among the elderly. High levels of interleukin-6, IL-1, tumor necrosis factor-α, and C-reactive protein have been linked to an increased risk of morbidity and mortality in the elderly^9^. Chronic d-galactose treatment in rats induced neuronal apoptotic signaling pathways activation and neuroinflammation in the cerebral cortex and hippocampus^1^.

Aging results in a decline in Brain-derived neurotrophic factor (BDNF) which is a crucial protein engaged in plastic changes associated to learning and memory as well as a vital mediator for neuronal proliferation and integrity^10^.

The discovery of novel compounds delaying aging and supporting cognitive performance has become one of the greatest challenges. Metformin quickly penetrates the blood–brain barrier and builds-up in a number of brain areas, including the pituitary gland and hippocampus^11^. It has been demonstrated that long-term metformin administration reduces the onset of age-related pathological complications and lengthen longevity in humans^12^. Furthermore, metformin has been shown to improve cognitive impairment and behavioral disorders like anxiety in both diabetic and non-diabetic individuals^13,14^ and patients with neurodegenerative diseases^15^. However, the underlying mechanisms of metformin's effects in improving age-related neurocognitive impairment are complicated and yet not entirely understood.

The neuroprotective effects of metformin may be primarily attributable to the activation of the AMP-Activated Protein Kinase (AMPK) signaling pathway^11^. AMPK activation up-regulates the expression of BDNF, which has a critical role in synaptic transmission and memory consolidation^10^. In addition to this well-established property of AMPK activation, previous results have demonstrated that metformin can enhance memory through the restoration of oxidative stress, decrement of neuroinflammation, and inhibition of apoptosis, which may expand its clinical indications in the area of neurodegenerative disorders^14^. Metformin is a strong activator of the phosphatidylinositol 3‑kinase/protein kinase B (PI3K/Akt) pathway which is an important critical signaling pathway in inhibiting apoptosis and promoting cell survival^16^.

Taking into consideration metformin's benefits, which include it's safety, affordability, and usability; we studied the neuroprotective effects of metformin administration against neurocognitive impairment caused by d-galactose-induced aging in rats. Additionally, the underlying molecular mechanisms of these protective effects were invistigated.

## Materials and methods

### Animals

Thirty Wister albino male rats weighing 200–250 grams were used in the experiment after obtaining the necessary approvals from the Research Ethical Committee in the Faculty of Medicine, Menoufia University, Egypt (registration No. 10/2022 PHYS 9). Experimental procedures followed ARRIVE guidelines. The rats were housed in wire mesh cages (80 × 40 × 30 cm). All animals were provided full access to food and water during the study period after conditioning them for 2 weeks at constant environmental conditions and 12:12-h light/dark cycle.

### Experimental design

The animals were divided randomly into three groups (10/group):

1. Control group: Received saline solution via oral gavage.
2. d-galactose (D-gal) group: Received d-galactose (Sigma-Aldrich St. Louis, USA) dissolved in a saline solution in a dose of 100 mg/kg body weight via gastric lavage for eight consecutive weeks^17^
3. d-galactose + Metformin (D-gal + Met) treated group: Received d-galactose (by the same dose in the D-gal group) + metformin (Sigma-Aldrich St. Louis, USA) dissolved in a saline solution in a dose of 200 mg/kg/day via gastric lavage for eight consecutive weeks^16^

Following the completion of eight weeks, a neurobehavioral assessment of all rats was done. Thereafter, rats were anesthetized and sacrificed by cervical elongation and dislocation. The brain was extracted and washed with phosphate buffer saline (pH 7.4). The left hemisphere was weighed and divided into two halves; one of them was used for biochemical analysis and the other half was used for RT-PCR studies. The right hemisphere was fixed in a 10% formalin saline for histopathological and immunohistochemical assessment of the hippocampal tissues.

### Neurobehavioral tests

#### Novel object recognition

The novel object recognition test was used to assess the rats’ capacity to recognize a new object in a familiar setting. Each rat had three days of testing, which included three phases: habituation, training, and testing. Rats were placed into an open-field apparatus (50 cm × 50 cm × 40 cm) during the habituation phase, and they were given ten minutes for adaptation without any items. Each rat was kept in a chamber with two identical objects for five minutes during the training phase. After twenty-four hours, each rat was placed in the chamber for five minutes during the test phase, which involved the replacement of one of the old objects with a new one. The stopwatch was used to time the duration of object exploration. The discrimination index [= (novel object exploration time–familial object exploration time)/total exploration time × 100%] is used to assess rats' cognitive performance. Alcohol was used to clean the open field and the objects between rats^18^.

#### The Morris water maze

The Morris water maze was used to examine the impact of metformin on spatial learning and memory. A pool was made of stainless steel, had a diameter of 210 cm and a height of 50 cm including a submerged escape platform 1 cm below the water's surface. The water was maintained at 24 ± 1 °C. The acquisition task was tested over the course of five days of testing with four trials each day. The time needed to get to the secret platform during each trial was recorded as escape latency. A maximum of 60 seconds were given to the rats to locate the covert platform. A maximum time limit of 60 s was assigned, and the rat was manually guided to the hidden platform. A single probe trial was conducted 24 h after the last trial of the fifth day. The platform was removed and the rat was placed into the pool from the quadrant opposite to the training quadrant. Then, the rat was allowed to freely swim for 60 s. The time spent in the target quadrant was recorded^19^.

#### The elevated plus-maze (EPM) test

EPM is an established technique for evaluating anxiety-like behavior in rats. It was made of wood and consisted of open and closed arms (50 cm in length × 10 cm in width). The two closed arms were enclosed by 40 cm high walls. The arms were attached by a central square platform (10 × 10 cm). The apparatus was 50 cm above the floor. Each rat was placed in the center of the apparatus facing a closed arm and allowed to move freely for five minutes. The time spent in the open and closed arms was recorded. Also, open arms entries were recorded^20^. We also measure the Transfer Latency (TL) which used to assess memory and learning. On the first day (the acquisition session), each rat was exposed to EPM for 90 s. Time taken by the rat to reach the closed arm was recorded as the TL. Rats that failed to enter in closed arms in 90 s were excluded from the study. On the second day (the retention session), each rat was put into the open arm and the TL was recorded for a maximum of 90 s^21^.

### Biochemical analysis

Brain tissue was perfused with PBS solution, then homogenized in a 5 ml cold buffer per gram tissue. Centrifugation of the brain tissue was done at 4000 rpm for 15 minutes, then the supernatant was removed and stored at − 80 °C until measurements of Malondialdehyde (MDA) and Superoxide Dismutase (SOD) by using the conventional colorimetric (QuantiChrom™, BioAssay Systems, USA), serum Tumour Necrosis Factor (TNF-α), Interleukin 10 (IL-10), and Brain-Derived Neurotropic Factor (BDNF) by using ELISA kit (Quantikine, Abcam company, Cambridge, UK) according to the manufacturer’s instructions.

### Quantitative assay of AMPK and PI3K genes expression using reverse transcriptase polymerase chain reaction technique (RT-PCR)

Brain tissues were prepared for total RNA isolation using Qiagen RN easy plus Universal Kit from the USA. Then, RNA quality and purity were assured. RNA was stored at − 80 °C till use. Then, the first step was cDNA synthesis using QuantiTect Reverse Transcription Kit, Qiagen from the USA, using Applied Biosystems 2720 thermal cycler (Singapore) for only one cycle. GAPDH primers were used in RT-PCR reactions as an RNA loading control. The second step was cDNA amplification: cDNA was used in SYBR green-based quantitative real-time PCR for Relative Quantification (RQ) of AMPK and PI3K genes expression by SensiFASTTMSYBR Lo-ROX Kit, USA, using the following designed primers (Midland, Texas): The forward primer for AMPK was (TGCGTGTACGAAGGAAGAATCC). And the reverse primer was (TGTGACTTCCAGGTCTTGGAGTT). The forward primer for PI3K was (AGCTGGTCTTCGTTTCCTGA), and the reverse primer was (GAAACTTTTTCCCACCACGA). Finally, data analysis with the Applied Biosystems 7500 software version 2.0.1 was done. The RQ of AMPK and PI3K genes expression was performed using a comparative ∆∆Ct method, where the amount of the target genes (AMPK and PI3K) mRNA is normalized to an endogenous reference gene (GAPDH) and relative to a control.

### Histopathological assessment of the brain tissue

#### Histological examination

Brain tissue was fixed in a 10% formalin solution, embedded in paraffin, and serial coronal sections of 5 μm thickness were obtained. Sections of the brain hippocampus were then stained with Hematoxylin and Eosin (H&E) for examination with a light microscope.

#### Immunohistochemical staining of the hippocampus

Immunohistochemical staining of caspase-3 (CAT number: ab32351, Abcam company 152 Grove Street Waltham, M02453, USA) and synaptophysin (CAT number: ab32127, Abcam company 152 Grove Street Waltham, M02453, USA) were performed by using rabbit monoclonal antibodies. For Bcl-2 (CAT number: ab59348, Abcam company 152 Grove Street Waltham, M02453, USA), it took place by using rabbit polyclonal antibodies. They were received in a single vial containing 1 ml of antibody. Anti-caspase-3, anti-synaptophysin, and anti-Bcl-2 were used in diluted quantities 1:50, 1:400, and 1:100 respectively. Sections were cut at 5 µm and stained by an automated LINK 48 immunostainer (Dako, Agilent Technologies Inc, Santa Clara, USA). For heat retrieval, citrate buffer was used by immunostainer. Slides were stained automatically by primary diluted antibodies. Positive control for the reaction was performed using specific paraffin-embedded sections of the normal human tonsil, human pancreas, and follicular adenoma for caspase-3, synaptophysin, and Bcl-2, respectively. Negative controls were made by substituting the primary antibodies with non-immune serum.

Assessment of histopathology and immunohistochemical markers (caspase-3, synaptophysin and Bcl-2) was done in different areas of the hippocampus including CA1,CA2 and CA3.

### Statistical analysis

Results are expressed as mean ± Standard Deviation (SD). Analysis of Variances (ANOVA) was used for statistical analysis of the different groups, using Origin^®^ software and the probability of chance (p values). P values < 0.05 were considered significant.

### Ethics approval and consent to participate

This work was approved by and in accordance with the guidelines of the Ethical Committee of the Faculty of Medicine, Menoufia University, Egypt.

## Results

### Neurobehavioral tests

**Figure 1:**
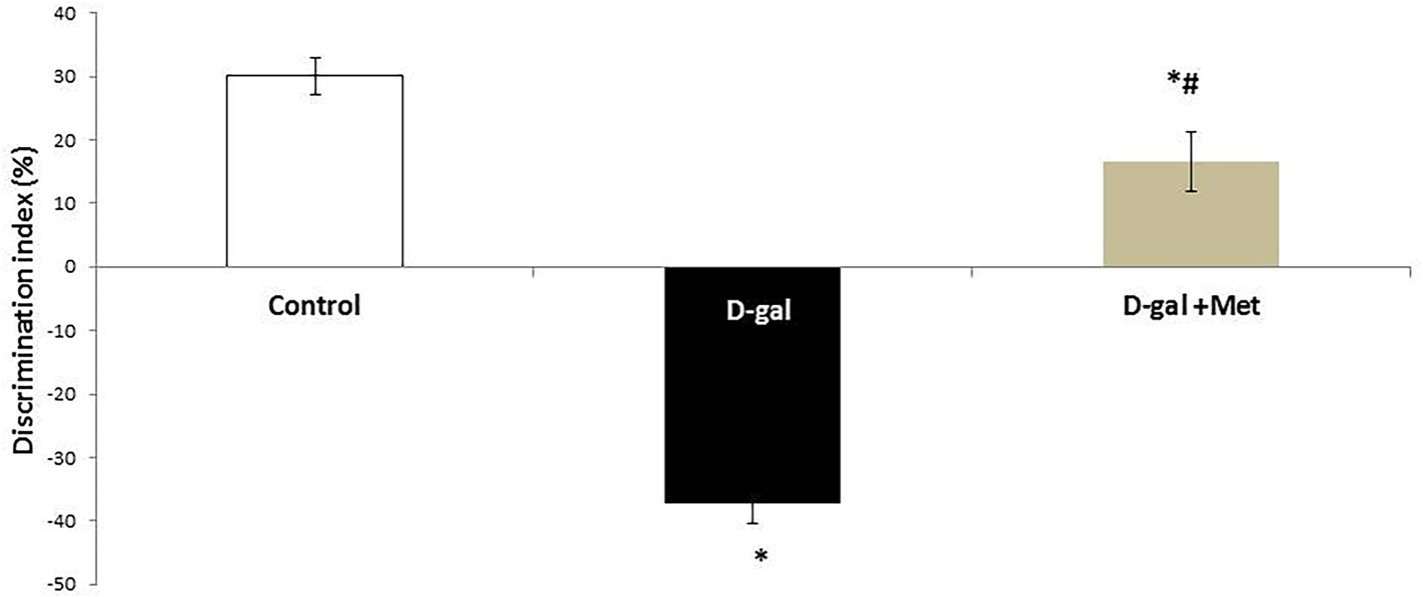
Effect of metformin on working memory and preference for novelty in d-galactose-induced aging rat model in novel object test (*significant when compared to control group, ^#^significant when compared to D-gal group). Data are shown as means + SD (n = 10). ANOVA was used to make group comparisons; Significance = P < 0.05).

Regarding the novel object test, the percentage of discrimination index was significantly lower in the D-gal group when compared with the control group (-37.16 ± 3.31% vs. 30.16 ± 2.92%, respectively, P < 0.05). The percentage of discrimination index was significantly higher in the D-gal + Met group (16.66 ± 4.67%, P < 0.05) when compared with the D-gal group. However, this percentage is still significantly lower when compared with the control group (Fig. 1).

**Figure 2:**
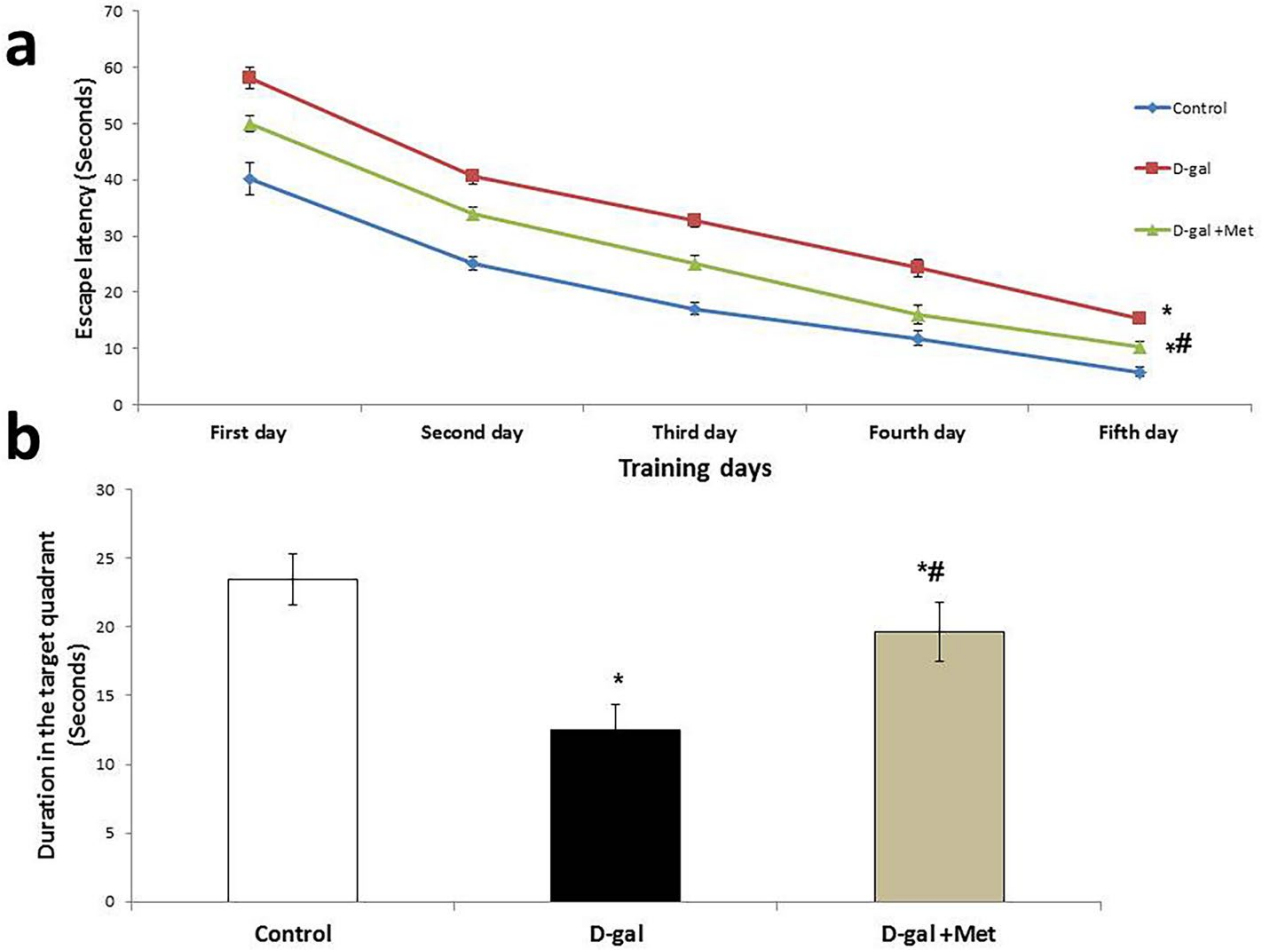
Effect of metformin on D-galactose-induced aging rat model in Morris water maze (*significant when compared to control group, ^#^significant when compared to D-gal group. Data are shown as means + SD (n = 10). ANOVA was used to make group comparisons; Significance = P < 0.05).

Regarding the Morris maze test, the mean value of the duration of escape latency was significantly higher in the first, second, third, fourth, and fifth days in the D-gal group when compared with the control group (58.16 ± 1.94, 40.62 ± 1.33, 32.75 ± 1.10, 24.37 ± 1.58, and 15.29 ± 0.98 s vs. 40.16 ± 2.92, 25.16 ± 1.15, 17.04 ± 1.08, 11.75 ± 1.31, and 5.83 ± 0.86 s, respectively, P < 0.05). The mean value of the duration of escape latency was significantly lower in the D-gal + Met group in the five training days (50 ± 1.41, 34.08 ± 1.15, 25.16 ± 1.31, 16.04 ± 1.75, and 10.25 ± 1.01 s, respectively, P < 0.05) when compared with the D-gal group. However, the mean values of the durations of escape latency in the five training days in the D-gal + Met group were still significantly higher when compared with the control group**.** The mean value of the duration that the rats spent in the target quadrant was significantly lower in the D-gal group when compared with the control group (12.50 ± 1.87 vs. 23.50 ± 1.87 s, respectively, P < 0.05). However, the mean value of the duration that the rat spent in the target quadrant was significantly higher in the D-gal + Met group (19.66 ± 2.16 s, P < 0.05) when compared with the D-gal group. However, its level is still significantly lower when compared with the control group (Fig. 2).

**Figure 3:**
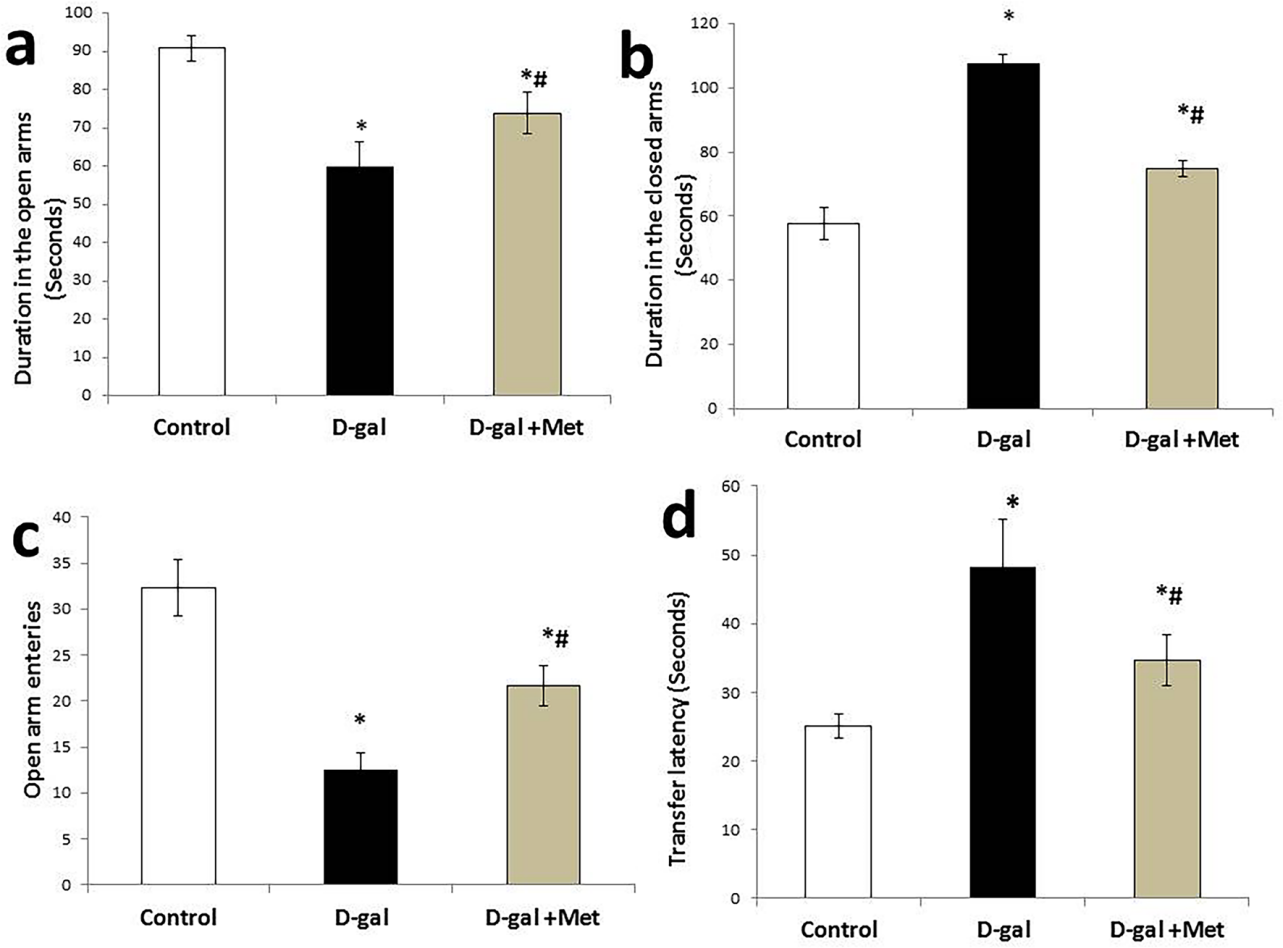
Effect of metformin on anxiety-like behavior in D-galactose-induced aging rat model in EPM test (*significant when compared to control group, ^#^significant when compared to D-gal group). Data are shown as means + SD (n = 10). ANOVA was used to make group comparisons; Significance = P < 0.05).

In the elevated plus-maze test, the mean value of the duration that the rats spent in the open arms was significantly lower in the D-gal group when compared with the control group (60 ± 6.54 s vs. 90.83 ± 3.48 s, respectively, P < 0.05). On the other hand, the mean value of the duration that the rats spent in the open arms was significantly higher in the D-gal + Met group (73.83 ± 5.41 s, P < 0.05) when compared with the D-gal group. However, its level was still significantly lower when compared with the control group. On the contrary**,** the mean value of the duration that the rats spent in the closed arms was significantly higher in the D-gal group when compared with the control group (107.33 ± 3.07 s vs. 57.66 ± 5.12 s respectively, P < 0.05). The mean value of the duration that the rats spent in the closed arms was significantly lower in the D-gal + Met group (74.66 ± 2.58 s, P < 0.05) when compared with the D-gal group. However, its level was still significantly higher when compared with the control group**.** The mean value of the number of open-arm entries was significantly lower in the D-gal group when compared with the control group (12.50 ± 1.87 vs. 32.33 ± 3.07, respectively, P < 0.05). On the contrary, the mean value of the number of open-arm entries was significantly higher in the D-gal + Met group (21.66 ± 2.16, P < 0.05) when compared with the D-gal group. However, its level is still significantly lower when compared with the control group. The mean value of the duration of the transfer latency was significantly higher in the D-gal group when compared with the control group (48.33 ± 6.88 s vs. 25 ± 1.87 s, respectively, P < 0.05). While the mean value of the duration of the transfer latency was significantly lower in the D-gal + Met group (34.66 ± 3.77 s, P < 0.05) when compared with the D-gal group. However, its level was still significantly higher when compared with the control group (Fig. 3).

### Biochemical tests

**Figure 4:**
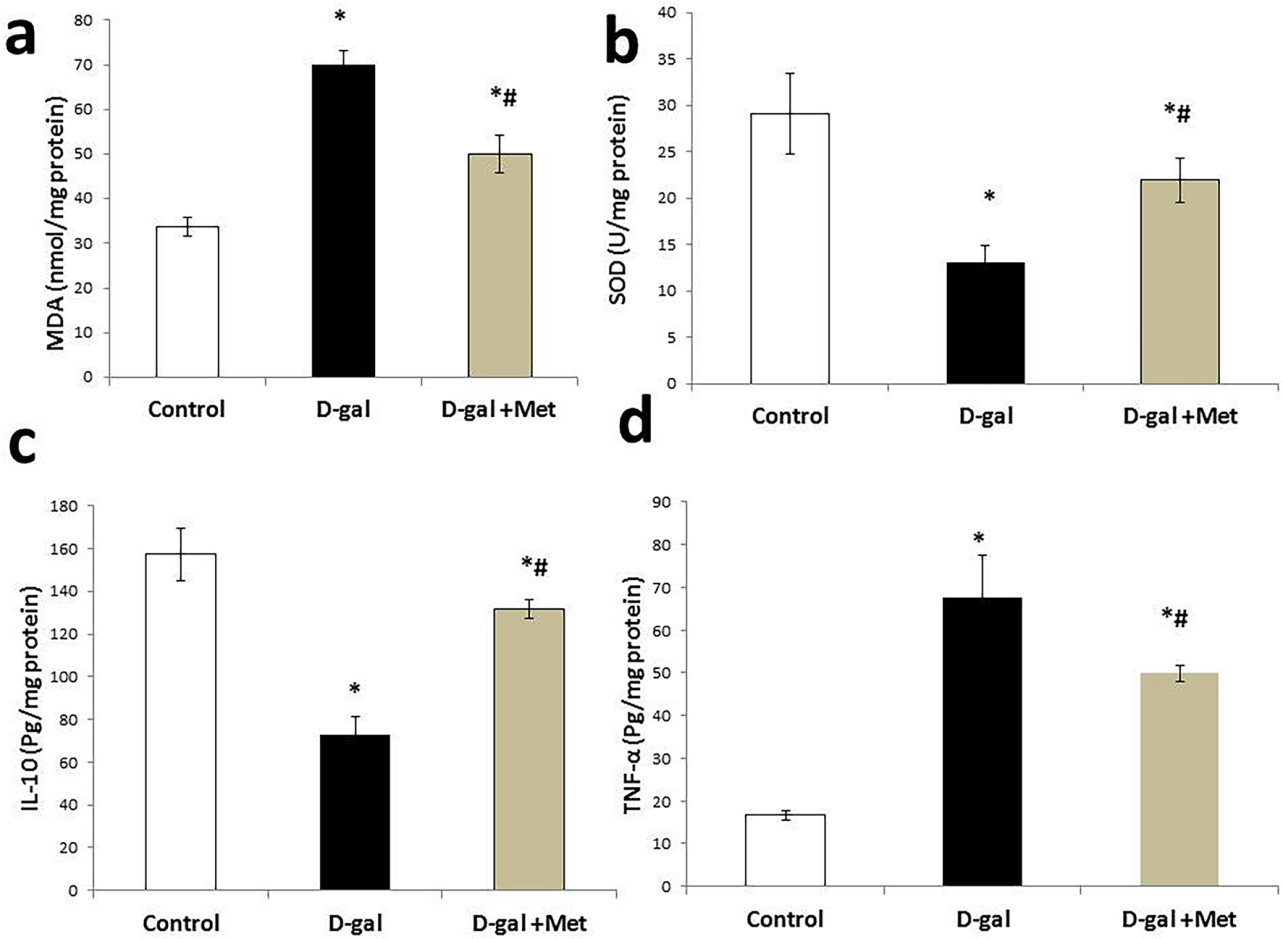
Metformin ameliorates the oxidative stress and inflammatory status in D-galactose-induced aging rat model (*significant when compared to control group, ^#^significant when compared to D-gal group). Data are shown as means + SD (n = 10). ANOVA was used to make group comparisons; Significance = P < 0.05).

The mean value of brain MDA level was significantly higher, while the mean value of brain SOD level was significantly lower in the D-gal group when compared with the control group (70 ± 3.23 nmol/mg protein and 13.05 ± 1.86 U/mg protein vs 33.76 ± 2.13 nmol/mg protein and 29.11 ± 4.34 U/mg protein respectively, P < 0.05). The mean value of brain MDA level was significantly lower, while the mean value of brain SOD level was significantly higher in the D-gal + Met group (50.02 ± 4.26 nmol/mg protein and 21.90 ± 2.33 U/mg protein, respectively, P < 0.05) when compared with the D-gal group. However, the mean value of MDA is still significantly higher and the mean value of SOD significantly lower when compared with the control group (Fig. 4a,b).

The mean value of brain TNF-α level was significantly higher, while the mean value of brain IL-10 level was significantly lower in the D-gal group when compared with the control group (67.50 ± 10.03 pg/mg protein and 72.91 ± 8.45 pg/mg protein vs. 16.61 ± 1.21 pg/mg protein and 157.23 ± 12.46 pg/mg protein, respectively, P < 0.05). The mean value of brain TNF-α level was significantly lower, while the mean value of brain IL-10 level was significantly higher in the D-gal + Met group (49.83 ± 1.89 pg/mg protein and 131.67 ± 4.54 pg/mg protein, respectively, P < 0.05) when compared with the D-gal group. But, the mean value of TNF-α was still significantly higher and the mean value of IL-10 significantly lower when compared with the control group (Fig. 4c,d).

**Figure 5:**
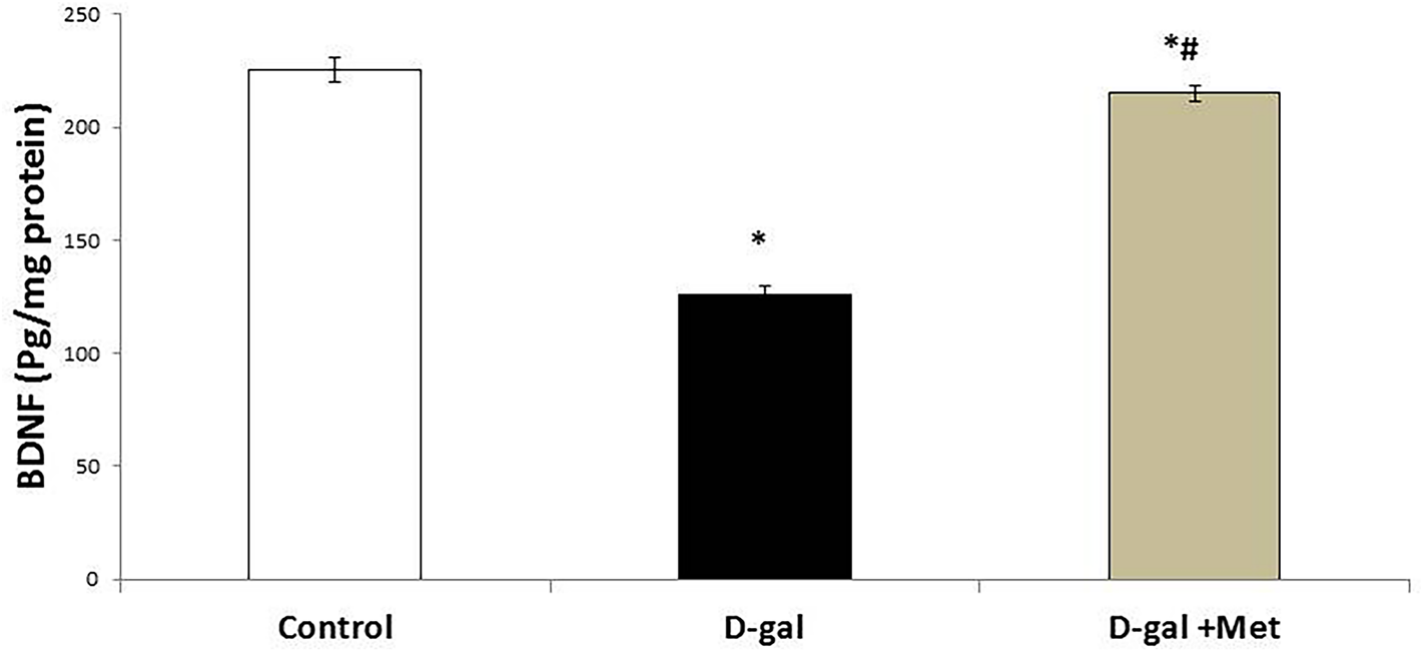
Metformin improves brain BDNF level in D-galactose-induced aging rat model. (*significant when compared to control group, ^#^significant when compared to D-gal group). Data are shown as means + SD (n = 10). ANOVA was used to make group comparisons; Significance = P < 0.05).

The mean value of brain BDNF level was significantly lower in the D-gal group when compared with the control group (125.72 ± 4.17 pg/mg protein vs. 225.20 ± 5.52 pg/mg protein, respectively, P < 0.05). The mean value of brain BDNF level was significantly higher in the D-gal + Met group (215.03 ± 3.65 pg/mg protein, P < 0.05) when compared with the D-gal group. However, its level is still significantly lower when compared with the control group (Fig. 5).

### Quantitative RT-PCR (qRT-PCR)

**Figure 6:**
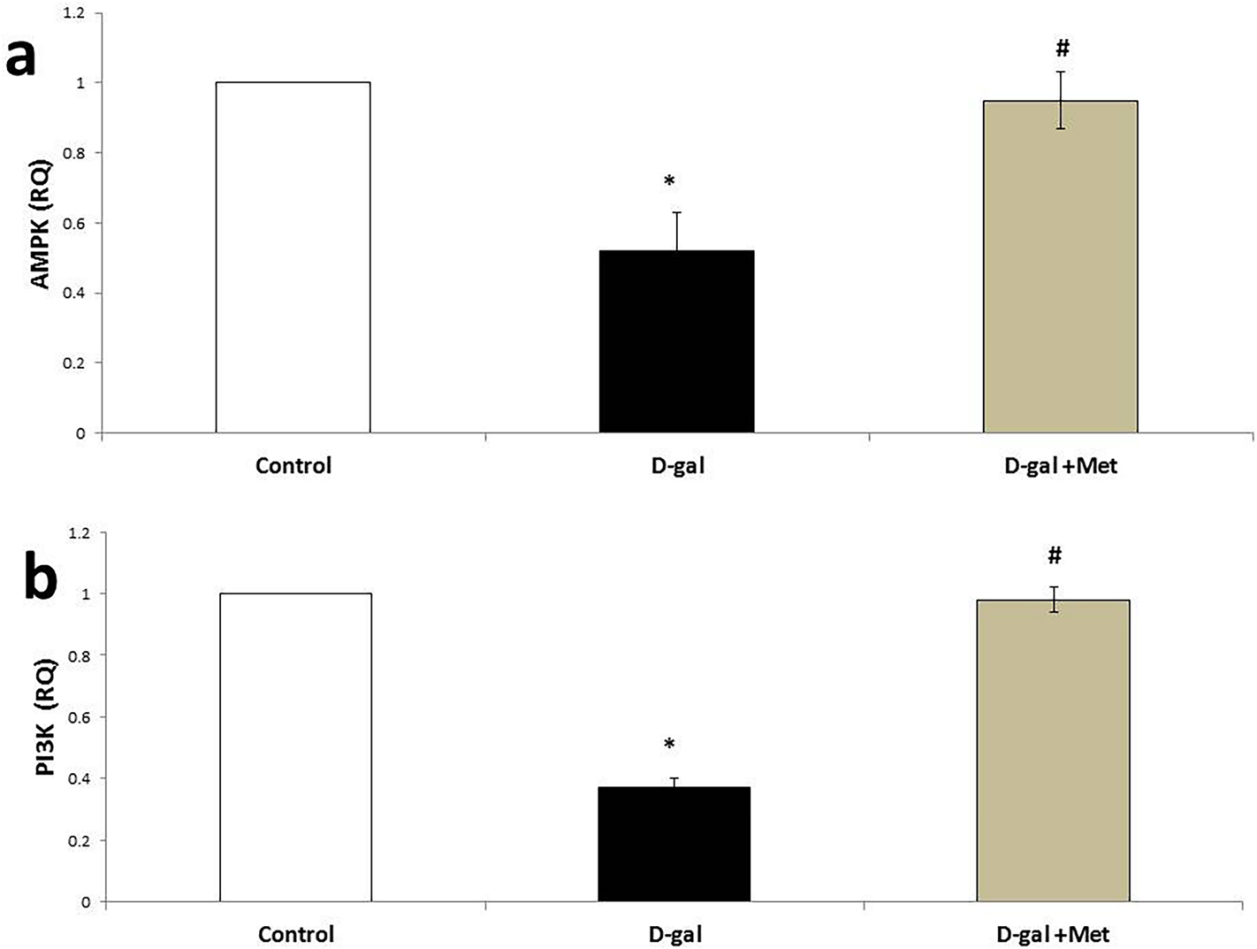
Gene modifying effect of metformin in D-galactose-induced aging rat model (*significant when compared to control group, ^#^significant when compared to D-gal group). Data are shown as means + SD (n = 10). ANOVA was used to make group comparisons; Significance = P < 0.05).

The expression of the AMPK and PI3k genes (0.52 ± 0.11and 0.37 ± 0.03, respectively, P < 0.05) was significantly down-regulated in the D-gal group when compared with the control group (1). However, the expression of AMPK and PI3k genes was significantly up-regulated in the D-gal + Met group (0.95 ± 0.08 and 0.98 ± 0.04, respectively, P < 0.05), when compared with the D-gal group, but it was insignificantly changed (P > 0.05) when compared with the control group (Fig. 6).

### Histopathology

**Figure 7:**
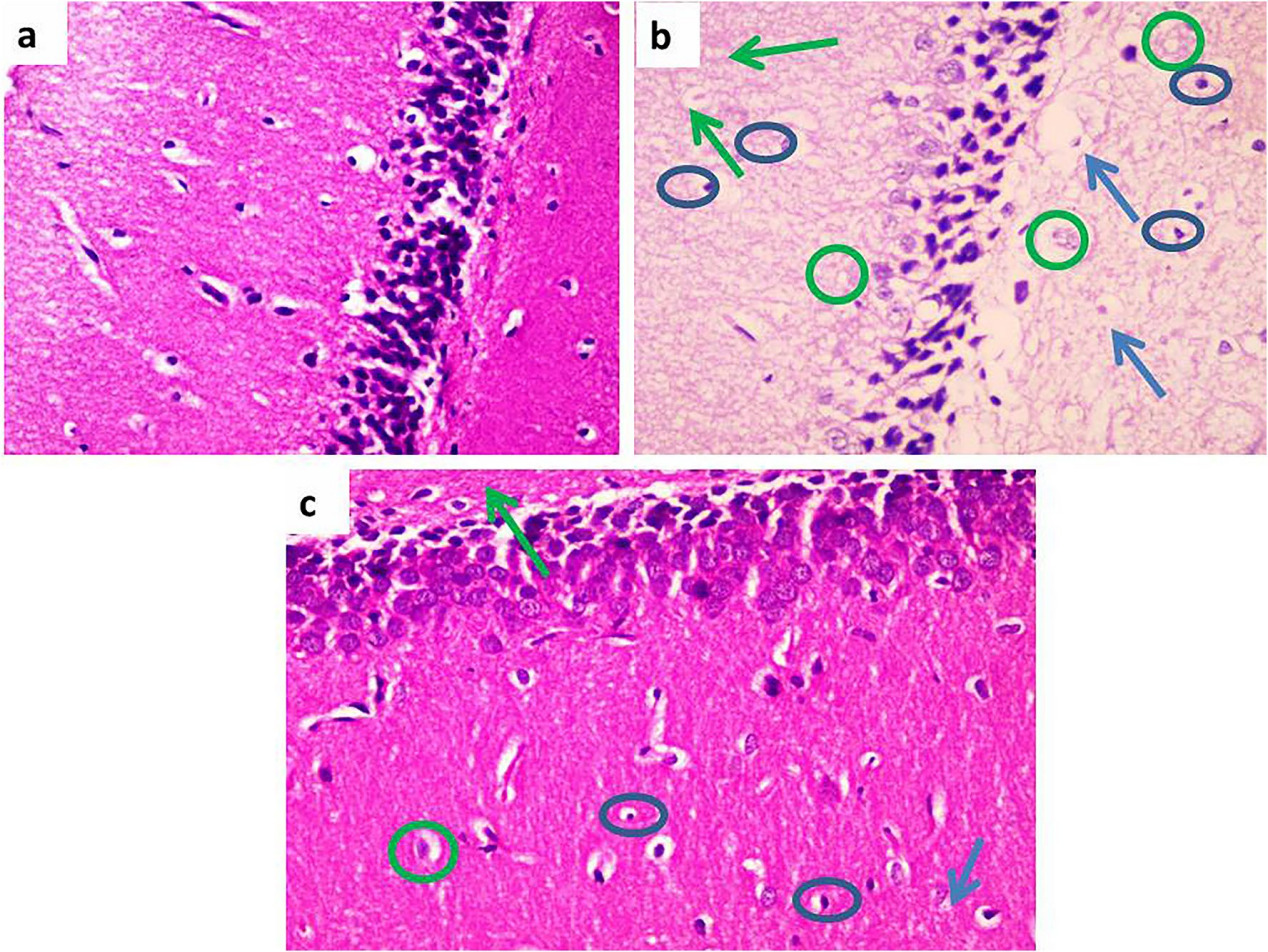
Effect of metformin on hippocampal tissue structure in D-galactose induced-aging rat model. (**a**) Section of the hippocampal tissue in the control group showed unremarkable pathological changes. (**b**) Section of the hippocampal tissue in the D-gal group showed many neuronal degeneration (green circles), and apoptotic bodies (blue circles) with massive oedema (blue arrows) and gliosis (green arrows). (**c**) Section of the hippocampal tissue in the D-gal + Met group showed few neuronal degeneration (green circles) and few apoptotic bodies (blue circles) with mild oedema (blue arrows) and mild focal gliosis (green arrows). (H&E × 200 for A, B and C). (Number of rats = 10/group).

The H&E stain of hippocampal tissue in the control group revealed unremarkable pathological changes. The D-gal group showed much neuronal degeneration and apoptotic bodies with massive edema and gliosis. However, the D-gal + Met group sections showed few neuronal degenerations and few apoptotic bodies with mild edema and mild focal gliosis (Fig. 7).

### Immunohistochemistry

**Figure 8:**
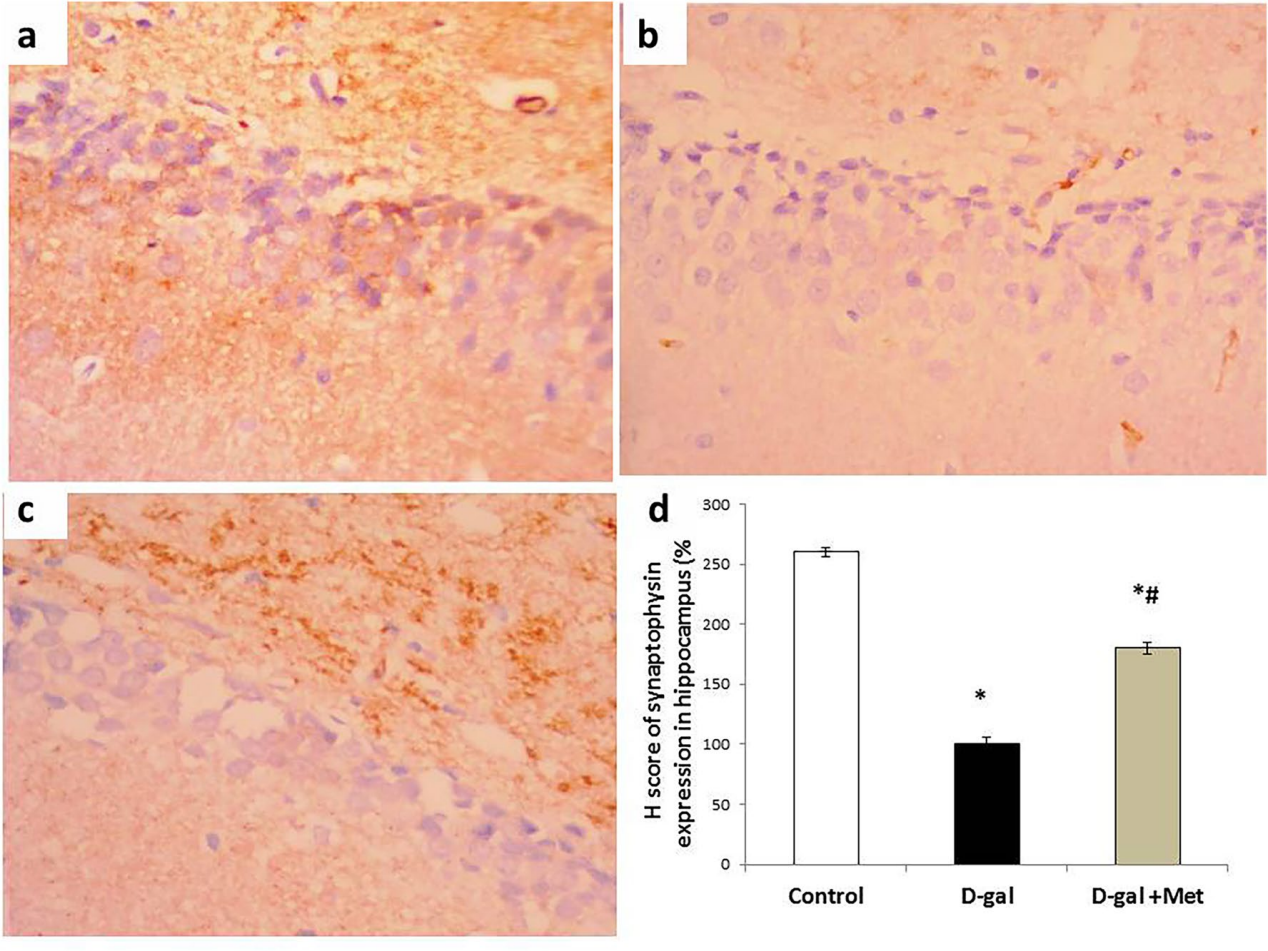
Synaptophysin immunostaining in hippocampal tissues of the studied groups. (**a**) Section of the hippocampal tissue in the control group showed intense cytoplasmic expression of synaptophysin in neural cells. (**b**) Section of the hippocampal tissue in the D-gal group showed mild cytoplasmic expression of synaptophysin in neural cells. (**c**) Section of the hippocampal tissue in the D-gal + Met group showed moderate cytoplasmic expression of synaptophysin in neural cells (Synaptophysin × 200 for A, B and C). (**d**) Represents the percentage of synaptophysin expression in the above-mentioned groups (*significant when compared to control group, ^#^significant when compared to D-gal group). Data are shown as means + SD (n = 10). ANOVA was used to make group comparisons; Significance = P < 0.05).

The immunohistochemical results revealed that the H score value of synaptophysin expression in hippocampal tissue was significantly lower (P < 0.05) in the D-gal group when compared with the control group (100 ± 5.78 vs. 260 ± 4.03). However, the expression of synaptophysin (180 ± 5.26) in hippocampal tissue in the D-gal + Met group was significantly higher when compared with the D-gal group. However, it was still significantly lower when compared with the control group (Fig. 8).

**Figure 9:**
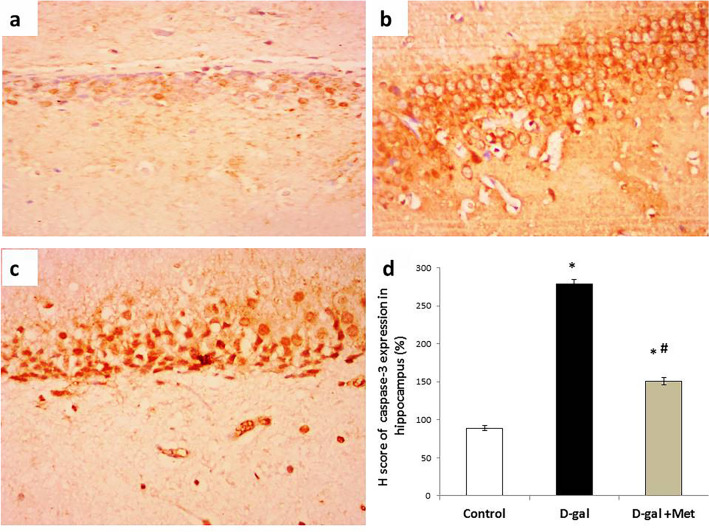
Caspase-3 immunostaining in hippocampal tissues of the studied groups. (**a**) Section of the hippocampal tissue in the control group showed mild cytoplasmic expression of Casepase-3 in glial and neural cells. (**b**) Section of the hippocampal tissue in the D-gal group showed marked cytoplasmic expression of synaptophysin in neural cells. (**c**) Section of the hippocampal tissue in the D-gal + Met group showed moderate nucleocytoplasmic expression of Casepase-3 in neural cells (Casepase-3 × 200 for A, B and C). (**d**) Represents the percentage of Casepase-3 expression in the above-mentioned groups (*significant when compared to control group, ^#^significant when compared to D-gal group). Data are shown as means + SD (n = 10). ANOVA was used to make group comparisons; Significance = P < 0.05).

The H score value of the caspase-3 expression in hippocampal tissue was significantly higher (P < 0.05) in the D-gal group when compared with the control group (279 ± 5.35 vs. 89 ± 3.54). On the other hand, the H score value of the caspase-3 expression in hippocampal tissue in the D-gal + Met group (151 ± 4.73) was significantly lower when compared with the D-gal group, but still significantly higher when compared with the control group (Fig. 9).

**Figure 10:**
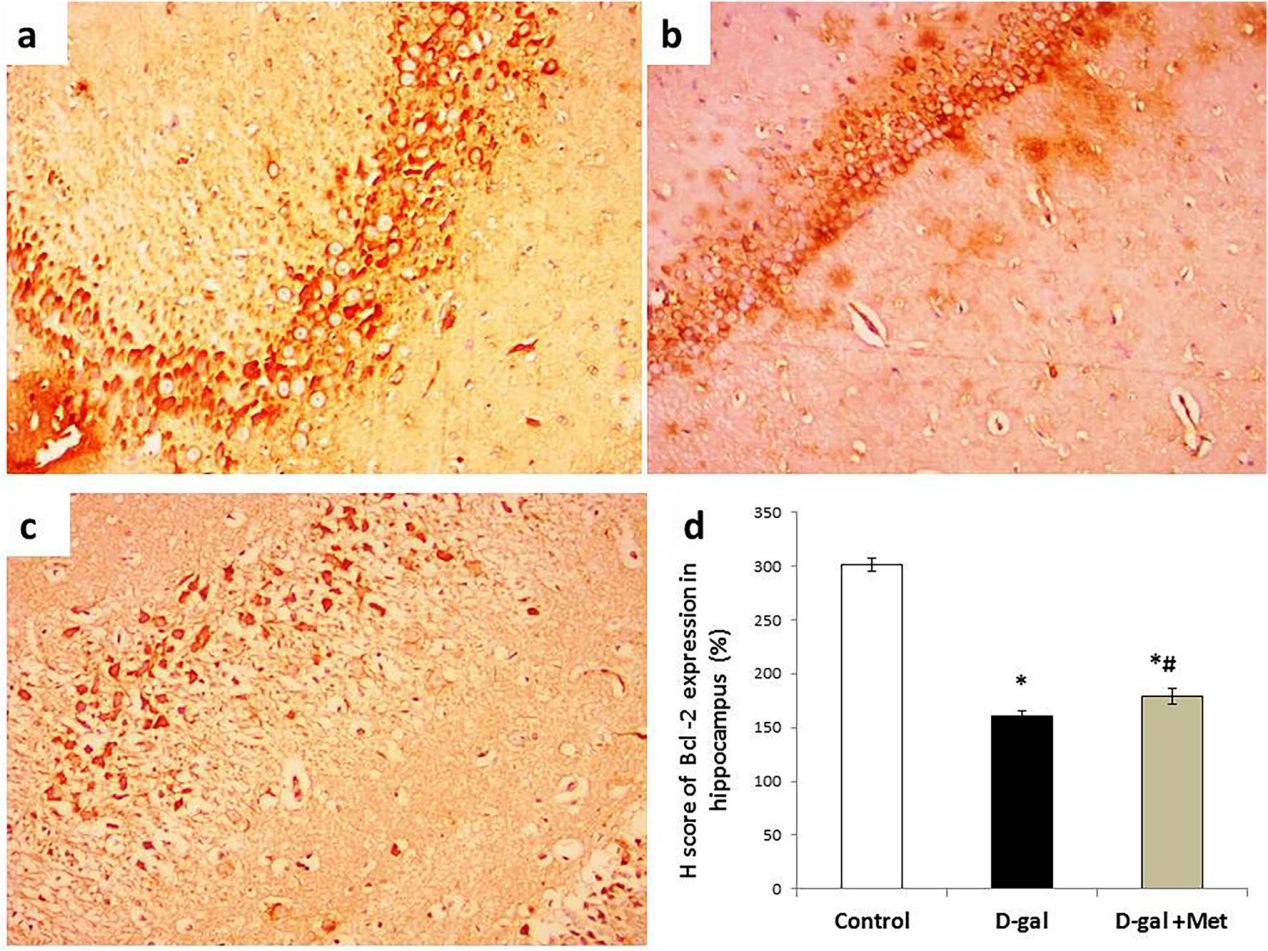
Bcl-2 immunostaining in hippocampal tissues of the studied groups. (**a**) Section of the hippocampal tissue in the control group showed intense cytoplasmic expression of Bcl-2 in neural cells. (**b**) Section of the hippocampal tissue in the D-gal group showed mild focal cytoplasmic expression of Bcl-2 in neural cells. (**c**) Section of the hippocampal tissue in the D-gal + Met group showed moderate cytoplasmic expression of Bcl-2 in neural cells (Bcl-2 × 200 for A, B and C). (**d**) Represents the percentage of Bcl-2 expression in the above-mentioned groups (*significant when compared to control group, ^#^significant when compared to D-gal group). Data are shown as means + SD (n = 10). ANOVA was used to make group comparisons; significance = P < 0.05).

The H score value of Bcl-2 expression in hippocampal tissue was significantly lower (P < 0.05) in the D-gal group when compared with the control group (161 ± 4.36 vs. 301 ± 6.11). While the H score value of Bcl-2 expression in hippocampal tissue in the D-gal + Met group (179 ± 7.48) was significantly higher when compared with the D-gal group, but it was still significantly lower when compared with the control group (Fig. 10).

## Discussion

The increase in average life expectancy increases the risk of illness in the elderly, especially in the cognitive arena which might be, at least in part, due to neuronal loss in the brain. It is well accepted that the long-term injection of d-galactose contributes to the aging progress and slight neuronal damage and memory deficits. Since D-gal-induced aging is accompanied by neurodegeneration, it could be an ideal model for studying the molecular mechanisms involved with age-associated neurodegeneration and for testing new therapeutic approaches^22^.

Our study showed that chronic administration of D-gal impaired novelty-induced exploratory behaviors and working memory in rats. This was obvious in the results of the novel object, Morris water maze, and elevated plus-maze tests. These results were in line with other reported studies^23^. On the other hand; treatment with metformin improved the working memory and preference for novelty when compared with the D-gal group. Supporting our results, a previous study reported that metformin improved short-term memory in streptozotocin-induced diabetic mice^24^. It is well documented that aging is associated not only with a decline in cognitive functions but also with emotional changes including anxiety-like behavior which results in functional impairment^25^. In the present study, EPM test results showed increased anxiety-like behavior in the D-gal group when compared with the control group. While treatment with metformin attenuated anxiety-like behavior.

The present study revealed that neurobehavioral changes caused by oral administration of D-gal were accompanied by disturbances in brain oxidative stress markers. The mean value of MDA level in brain tissue homogenate was significantly higher, while the activity of the antioxidant enzyme SOD was significantly lower in the D-gal group when compared with the control group. Similar results were previously reported^23,26^. D-gal interacts with various free amines in the protein architecture via non-enzymatic glycation, resulting in the generation of advanced glycation products. This results in the generation of ROS which increases brain aging via oxidative damage to DNA, lipids, and proteins. Memory loss and learning impairment induced by chronic administration of D-gal could be attributed to the generation of free radicals, resulting in impairment of neurogenesis and, ultimately, neurodegeneration^27^. Moreover, brain neurons are more susceptible to oxidative stress due to the presence of high lipid content and higher oxygen consumption^26^.

On the contrary, metformin administration significantly restored the redox balance. Evidence demonstrated that metformin could inhibit the Mitochondrial Permeability Transitional Pore (mPTP) and reduce ROS production and lipid peroxidation^28^. Therefore, this indicates that the anti-aging effect of metformin might be possibly mediated by its antioxidative defense.

Besides oxidative stress, inflammation is also an important aging-inducing mechanism of D-gal treatment. D-gal promotes the generation of ROS that activate inflammatory pathways^3^. Chronic neuroinflammation and secretion of pro-inflammatory cytokines is a hallmark of aging^29^. There is an agreement that inflammatory cytokines are involved in oxidative stress. TNF-α, IL-6, and IL-1β are essential in the development and progression of oxidative stress. These cytokines are associated with ROS and activate Nuclear factor-κB (NF-κB) to translocate to the nucleus and regulate the expression of pro-inflammatory genes such as iNOS and COX2, which are involved in inflammatory and immune responses, and consequently leads to fibrosis, apoptosis, and acute phase responses that cause organ damage^30^. The aforementioned data was consistent with our results, as the level of TNF-alpha was significantly higher, and IL-10 was significantly lower in the D-gal group compared with the control group.

On the other hand, treatment with metformin resulted in a significant reduction in the inflammatory markers when compared with the D-gal group. The anti-inflammatory effects of metformin could be attributed to the activation of AMPK signaling, which suppresses inflammatory reactions via the inhibition of NF-κB^29^. Also, it decreased pro-inflammatory cytokines production and microglial activation in the brain which, in turn, decrease oxidative stress^30^.

To further understand the molecular mechanisms underlying metformin actions, we examined the protein expressions of AMPK and PI3k. Our study revealed that the expression of AMPK was significantly down-regulated in the D-gal group when compared with the control group, while its level was significantly up-regulated in the metformin-treated group when compared with the D-gal group. Previous studies reported that the AMPK activation and AMPK responsiveness decrease with age, which may explain the altered metabolic regulation, resulting in reduced autophagy and an increase in oxidative stress^31^. Metformin were found to activate AMPK by increasing the phosphorylation of AMPKα at Thr-172^32^. AMPK is the primary target of metformin for its protective function against cognition disruption. Ghadernezhad et al. reported that metformin-induced activation of AMPK led to the activation of the BDNF/P70S6K pathway in hippocampal neurons to enhance the formation of memory in passive avoidance task in a global cerebral ischemia/reperfusion rat model. BDNF activation has a vital role in the regulation of neurocognitive functions like learning, memory, synaptic transmission, and plasticity^33^. Several studies demonstrated that AMPK could act as a modulator of Long-Term Potentiation (LTP), and it is required for memory formation^34^. Therefore, metformin-induced memory improvement in our study could be mediated through AMPK signaling activation.

Also, activation of AMPK signaling suppresses inflammatory reactions via activating SIRT1^29^, stimulating FOXO proteins^35^ and inhibiting ER stress, and reducing oxidative stress. All these mechanisms will subsequently repress NF-κB signaling and neuroinflammation associated with aging^36^. In addition to its anti-inflammatory effects, AMPK is involved in various activities, including angiogenesis, autophagy enhancement, and mitochondrial protein induction^16^.

Although the AMPK-dependent protective roles in different contexts have been reported, the AMPK-independent manners of metformin are less studied. The present study revealed that there was significant down-regulation of the PI3K gene in the D-gal group when compared with the control group, while metformin co-administration led to the up-regulation of its level when compared with the D-gal group. PI3K/Akt is one of the important signaling pathways in cell apoptosis prevention. Activation of PI3K can be followed by Akt1 phosphorylation. Akt1 can inhibit the phosphorylation of JNK3 that promotes the activation of c-Jun. C-Jun is a protein that can induce the expression of apoptotic proteins^37^. Thus, Akt1 activation will result in the inactivation of JNK3 and c-Jun, and, then the proportion of cell survival is improved. Therefore, we explored the effects of metformin on cellular apoptosis by studying the immunohistochemical reaction of the apoptotic markers caspase-3 and Bcl-2. This study revealed an increment in the expression of the apoptotic marker caspase-3 with a reduction of the expression of the anti-apoptotic marker Bcl-2 in the hippocampus of the D-gal group when compared with the control group. Supporting our results, previous studies confirmed that mitochondrial ROS induce the activation of a large number of mitochondrial apoptotic proteins, leading to cellular apoptosis and organ damage^38^. Many apoptotic proteins are closely related to anti-apoptotic proteins in the aging induced by the injection of D-gal^39^. Caspase-3 is known to be a key factor of apoptosis in mammals^40^. The Bcl-2 protein is a key player in the inhibition of apoptosis. It is a known factor in cell aging, and its overexpression can effectively prevent the apoptosis induced by free radicals^41^. It is commonly believed that Bcl-2 acts downstream of caspase-3 activation and, thus, apoptosis is inhibited^42^. As shown in our results, metformin administration significantly decreases the Caspase-3/Bcl-2 ratio. These results were in accordance with other studies that suggest that activation of AMPK by metformin up-regulates the Bcl-2 protein expression so it protects against apoptotic cell death induced by D-gal and increases neuronal viability^43^.

BDNF is an interesting molecular candidate that could help establish a link between molecular and biochemical alterations and memory deficits associated with aging. Optimal cognitive function is linked to efficient neuronal plasticity. Memory deficits associated with aging might be coupled to alterations in the expression and regulation of plasticity-related proteins such as BDNF which is an important neurotrophic factor. It has been demonstrated that reduction of BDNF leads to neuronal atrophy and death^44^.

The present study revealed that BDNF level in the D-gal group was significantly lower when compared with the control group. A decrease in BDNF and/or its receptors in aging animals was evident in previous studies^45^. Mizisin et al. suggested that galactose metabolism by aldose reductase influenced axonal function and structure by altering the production of nerve and muscle BDNF^46^. On the contrary, treatment with metformin was associated with a significant increase in BDNF when compared with the D-gal group. Our results were in agreement with previously published reports which demonstrated that metformin up-regulates BDNF via AMPK activation^47^.

Due to its critical role in LTP, BDNF has been postulated to be an essential part of the cellular mechanism supporting memory formation and maintenance by promoting synaptic consolidation. BDNF increases memory storage by favoring changes in spine morphology leading to the stabilization of LTP. BDNF can also increase the number, size, and complexity of dendritic spines. Furthermore, BDNF increases neurogenesis through changes in cell proliferation^10^. The binding of BDNF to TrkB receptors induces PI3K activity which inhibits apoptosis and promotes cell survival^22^.

Taken together, our study suggests that the anti-aging effects of metformin in improving neurocognitive impairment could be, at least in part, due to the activation of AMPK/BDNF/PI3K pathway.

Hippocampus plays an important role in learning and memory consolidation as well as in behaviors and mood regulation, and where both functional and structural plasticity occur well into adulthood. Previous studies reported that hippocampus undergoes several structural changes both grossly and at the cellular level with aging^48^*.* H & E study of the hippocampus in the D-gal group showed severe neuronal degeneration with multiple apoptotic bodies and gliosis. This result could be explained by the deleterious effects of d-galactose in the induction of oxidative stress, inflammation, and apoptosis. Additionally, we evaluated the expression levels of a synaptic marker protein (synaptophysin) in the hippocampus. Synaptophysin is a marker of synaptic plasticity. It is used as a specific marker for the presynaptic terminal, and its level is related to the synaptic density. Our results showed that the expression of synaptophysin was significantly lower in the D-gal group when compared with the control group. These results were in line with previously reported studies that demonstrated that D-gal-induced synaptogenesis impairment in the hippocampus^3^. However, metformin co-treatment with D-gal restored the synaptophysin expression and hippocampal tissue structure to levels close to their respective control levels. Evidence has shown that metformin promotes rodent and human neurogenesis and enhances spatial memory formation^24,41^. The increment in synaptic density and neurogenesis in the hippocampus goes hand in hand with the improvement in the neurobehavioral tests' results in this group. This could be explained by the antioxidant, anti-inflammatory, and antiapoptotic effects of metformin, which are mediated by activation of AMPK/BDNF/PI3K signaling pathway.

## Conclusions

Our findings support the use of D-gal in the rat model to carry out aging-related studies. We concluded that metformin could alleviate memory impairment and cognitive deficit caused by aging. The mechanisms likely involved are amelioration of neuro-inflammation, attenuation of oxidative stress, enhancement of the expression of the anti-apoptotic protein Bcl-2, as well as the promotion of neurogenesis and synaptic plasticity. We believed that these mechanisms could be mediated via activation of the AMPK/BDNF/PI3K pathway. To the best of our knowledge this is the first study demonstrate the action of metformin on improving cognitive impairment in aged rats via activation of this pathway. Therefore, our findings suggest that metformin is a useful anti-aging agent.

## Data Availability

All the data generated or analysed during this study are included in this published manuscript.

## Abbreviations

D-gal: d-galactose
ROS: Reactive oxygen species
IL-1: Interleukin-1
IL-1β: Interleukin-1β
IL-6: Interleukin-6
IL-10: Interleukin-10
TNF-α: Tumor necrosis factor
BDNF: Brain derived neurotropic factor
AMPK: AMP-activated protein kinase
PI3K/Akt: Phosphatidylinositol 3‑kinase/protein kinase B
RT-PCR: Reverse transcriptase polymerase chain reaction
EPM: Elevated plus maze
TL: Transfer latency
MDA: Malondialdehyde
SOD: Superoxide dismutase
MPTP: Mitochondrial permeability transitional pore
H&E: Haematoxylin and eosin
iNOS: Inducible nitric oxide synthase
NF-κB: Nuclear factor-κB
LTP: Long term potentiation
